# Examining Demographic Factors, Psychosocial Wellbeing and Cardiovascular Health in Subjective Cognitive Decline in the Brain Health Registry Cohort

**DOI:** 10.14283/jpad.2024.39

**Published:** 2024-03-05

**Authors:** Rachana Tank, A. Diaz, M. T. Ashford, M. J. Miller, J. Eichenbaum, A. Aaronson, B. Landavazo, J. Neuhaus, M. W. Weiner, R. S. Mackin, J. Barnes, R. L. Nosheny

**Affiliations:** 1 Dementia Research Centre, UCL Queen Square Institute of Neurology, University College London, London, UK; 2 University of California, San Francisco Department of Radiology and Biomedical Imaging, San Francisco, CA USA; 3 VA Advanced Imaging Research Center, San Francisco Veteran’s Administration Medical Center, San Francisco, CA USA; 4 Department of Veterans Affairs Medical Center, Northern California Institute for Research and Education (NCIRE), San Francisco, CA USA; 5 Department of Psychiatry and Behavioural Sciences, University of California San Francisco, San Francisco, CA USA

**Keywords:** Subjective cognitive decline, psychosocial, cardiovascular, gender, race

## Abstract

**Background:**

Subjective cognitive decline (SCD) is defined as an individual’s perception of sustained cognitive decline compared to their normal state while still performing within boundaries for normal functioning. Demographic, psychosocial and medical factors have been linked to age-related cognitive decline, and Alzheimer’s dementia (AD). However, their relation to risk for SCD remains unclear. This study aims to identify demographic factors, psychosocial and cardiovascular health associated with SCD within the Brain Health Registry (BHR) online cohort.

**Methods:**

Participants aged 55+ (N=27,596) in the BHR self-reported SCD measured using the Everyday Cognition Scale (ECog) and medical conditions, depressive symptoms, body mass index, quality of sleep, health, family history of AD, years of education, race, ethnicity and gender. Multivariable linear regression was used to examine whether SCD was associated with demographic, psychosocial, and medical conditions.

**Results:**

We found that advanced age, depressive symptoms, poorer sleep quality and poorer quality of health were positively associated with more self-reported SCD in all models. No race or ethnicity differences were found in association with SCD. Males who reported alcohol and tobacco use or underweight BMI had higher ECog scores compared with females.

**Conclusion:**

In addition to well-established risk factors for cognitive decline, such as age, our study consistently and robustly identified a strong association between psychosocial factors and self-reported cognitive decline in an online cohort. These findings provide further evidence that psychosocial health plays a pivotal role in comprehending the risk of SCD and early-stage cognitive ageing. Our findings emphasise the significance of psychosocial factors within the broader context of cardiovascular and demographic risk factors.

**Electronic Supplementary Material:**

Supplementary material is available for this article at 10.14283/jpad.2024.39 and is accessible for authorized users.

## Introduction

### Subjective Cognitive Decline

**S**ubjective cognitive decline (SCD) refers to self-identified decline in cognitive abilities while still performing within normal boundaries for neuropsychological assessments (1). Individuals with SCD tend to score lower on neuropsychological assessments in comparison to the general population and have a higher incidence of future cognitive decline (2). SCD is considered to be a preclinical manifestation of Alzheimer’s Dementia (AD) and Mild Cognitive Impairment (MCI) (3). A growing body of evidence shows older adults with SCD present with more AD related biomarkers than the general population (3). Given that SCD and AD share risk factors, evaluating and defining participant characteristics associated with at risk states such as SCD are important for identifying those at risk of cognitive decline. The (39-item) Everyday Cognition Scale (ECog) is a widely used measure for assessing self-reported cognitive decline and is considered reliable, valid, and sensitive to early cognitive and functional changes observed in conditions such as AD (4, 5). Previous work in a demographically diverse cohort has supported the construct validity of the domains measured by ECog (6).

### Risk Factors for SCD

Advanced age, low educational attainment and gender are independent risk factors for SCD (7–11). Psychosocial factors such as self-reported quality of health, quality of sleep, number of depressive symptoms, and gender differences have also been associated with SCD, however, their combined contributions are not well understood (12–17). A report examining combined contributions by examining eight potential modifiable risk factors (high blood pressure, not meeting the aerobic physical activity guideline, obesity, diabetes, depression, current cigarette smoking, hearing loss, and binge drinking) found that older adults with SCD were more likely to have four or more risk factors than those without SCD (18). However, it is unclear whether these findings can be replicated in a remote, online registry, deviating from conventional cohort studies.

The health and integrity of the ageing brain are closely related to physical health, and cerebrovascular alterations have also long been recognised as a common comorbidity of abnormal cognitive decline in ageing (19–24). Studies in cardiovascular disease epidemiology have highlighted the role of demographic factors like education in the development of conditions and risk factors, such as high BMI, smoking, and high blood pressure (25, 26) illustrating the need to consider demographic context in cardiovascular health. Relationships among demographic, psychosocial and medical factors are complex (27), and it is important to consider that risk factors can vary significantly among different ageing racial and ethnic groups. For example, education levels and health condition prevalence exhibit variation among ethnocultural groups and socioeconomic positions (11). Gupta (2021) found that disparities exist in education, income, healthcare access and chronic conditions among Black and Hispanic individuals with SCD compared with those without SCD. Wooten et al. (2023), studying North Americans, found the highest prevalence of SCD among American Indian or Alaska Native adults (16.7%) with prevalence declining with increasing formal educational attainment. Similarly, psychological stressors have been shown to be unequally distributed across different racial and ethnic groups (12). Ferraro et al., (2023) found that the prognostic validity for subjective memory decline differs amongst race and ethnicity groups. Robinson et al., (2023) similarly found stronger associations of psychological factors with SCD among non-Hispanic Black and Hispanic White groups (28). Therefore, it is notable that many SCD studies neglect to consider race and ethnicity in their analyses or discussions. Race and ethnicity have not been examined as extensively as other demographics in the context of SCD, nor in relation to such demographics.

Similarly, existing literature also strongly supports the significant influence of sex and gender in disease pathologies and mechanisms. Sex differences have been observed in the incidence, prevalence, age of onset, clinical presentation, diagnostic criteria, and disease severity of many conditions (13, 29–34), with evidence that SCD measures may be a stronger predictor of incident dementia for women than for men (31). Women demonstrate a lower incidence of cardiovascular disease, including stroke, before menopause, followed by higher incidence rates later in life surpassing those of men (13, 34–36). Despite gender differences in risk factors affecting cognitive health, the investigation of potential variations in the presentation of SCD based on gender remains an understudied area in research.

### Brain Health Registry

The Brain Health Registry (BHR) is a University of California, San Francisco (UCSF) research study, online website and registry for recruitment, assessment, and longitudinal monitoring of cognition, function, and health in adult volunteers (37–39). BHR was established in 2014 with approval by the UCSF Institutional Review Board and has over 90,000 participants, who contribute through self-report questionnaires and cognitive assessments every six months. Inclusion criteria for enrolment require participants to be aged 18 or over. Unsupervised online self-report questionnaires include demographic information, an online adaptation of the self-report ECog, health-related questions, medical history, depressive symptoms, memory complaints, and family history of AD (34, 35).

### Study Rationale and Hypothesis

This study’s primary objective is to identify AD risk factors associated with self-reported SCD in older adults enrolled in a longitudinal, remote, digital research study. It seeks to replicate and expand upon previous findings by examining multiple risk factors simultaneously, considering gender-specific associations within cardiovascular health, and incorporating consideration of race and ethnicity. Based on past findings of SCD and AD risk factors, we hypothesise that (1) ECog scores differ between different groups by race and by ethnicity; (2) poorer psychosocial health, cardiovascular medical conditions, and risk factors for cardiovascular conditions are associated with greater SCD; (3) associations between cardiovascular conditions and ECog differ between males and females. By examining these hypotheses, we aim to refine the characterisation of SCD populations and provide insights into cognitive ageing at its earliest stages, which can provide evidence on the significance or relevance of several understudied characteristics and risk factors.

## Methods

### Study Sample

The Brain Health Registry began enrolment in 2014 and is an ongoing longitudinal cohort study at the University of California, San Francisco. For this analysis, BHR participants were excluded if they self-reported AD, Dementia, Frontotemporal Dementia, Lewy Body Dementia, MCI, Multiple Sclerosis, or traumatic brain injury (N=2,038). Participants aged 55+ (N= 27,596) in the BHR cohort self-reported SCD (ECog), medical history, and demographic factors. Individuals with incomplete data were excluded. Supplementary Table 3. Shows a comparison of BHR participants who were excluded (n=62,633) vs included (n=27,596) in this study.

### Everyday Cognition Scale

BHR participants completed an online adaptation of the Everyday Cognition Scale (ECog), a 39-question assessment measuring the self-reported functional change in capability to perform everyday tasks (5). The ECog asks whether independence in activities of daily living across six cognitive domains (memory, language, visuospatial abilities, planning, organisation and divided attention) have changed. The ECog questionnaire scale ranges from 1 to 4 on a Likert scale, ranging from “better or no change” to “consistently much worse”. Scores for each participant were calculated by taking the mean of ECog questions answered. Higher ECog scores indicate greater self-reported cognitive decline.

### Demographic Variables

Gender, years of education, ethnicity, race, quality of sleep, quality of health, family history of AD and weight/height for BMI calculation was self-reported by participants. Gender is included as 0 for males and 1 for females. Education is reported in years and can range from 6 years corresponding to the completion of elementary school to 20 years for a doctoral or professional degree.

Participants were asked to self-report ethnicity as “Latino”, “Not Latino” or “Decline to State” where those who declined to state were included as having a missing value. Participants were asked to self-report race; race category options included “African American/Black”, “Asian”, “White”, “Native American”, “Pacific Islander”, “Other” or “Decline to State”. Individuals who selected more than one race (N=1,272) were grouped as “More than one race identity”. Those who declined to state were included as having missing values. Race categories were then coded as a multilevel categorical variable where white was set as the reference value.

Family history of AD was self-reported as 0=no or 1=yes.

### Psychosocial Variables

Depressive symptoms were measured using the Geriatric Depression Scale (GDS) (42, 43) where the score was the sum of 14 yes or no questions that evaluate the presence of depressive symptoms (43). One question was excluded from GDS score calculation, “Do you feel you have more problems with memory than most?”, as this question may be confounded with SCD. Higher scores indicate worse depressive symptom severity.

Quality of Sleep and Quality of health were dichotomised into 0 = bad, 1 = good from the questionnaires original scale where self-reported options were 0 = very good, 1 = fairly good, 3 = fairly bad, 4 = very bad.

### Cardiovascular Conditions and Risk Factors

BHR participants completed a self-report questionnaire on their medical history. They were asked to indicate whether they currently have or have had certain conditions. «The conditions included heart disease, high blood pressure, high cholesterol and diabetes». Responses to these questions were coded as binary variables (0=no, 1=yes). Heart disease, high blood pressure, high cholesterol, and diabetes were considered cardiovascular (CVD) related conditions.

Participants were also asked about past or current smoking and separately about alcohol use, coded as binary variables (0=no or 1=yes indicating past or current smoking or alcohol use) which were considered as risk factors for cardiovascular health. Overweight and obese BMI were also considered as risk factors for cardiovascular health. BMI calculations derived from self-reported height and weight to categorise individuals into four groups: underweight BMI (<18.5), normal BMI (18.5–24), overweight BMI (25-29), and obese BMI (>30). Each combination of height and weight was assigned to one of four predefined categories. For combinations close to category boundaries, a conservative approach was employed, categorising them toward the “normal” BMI range.

We addressed potential issues of multicollinearity between cardiovascular predictors by calculating generalized variance inflation factors associated with these variables, and found no significant indication of multicollinearity induced variance inflation (GViF < 1.64) that warranted excluding any predictor variables.

### Statistical Analyses

The primary objective of the statistical analyses was to identify associations between ECog scores and demographic, psychosocial and cardiovascular risk factors, as well as gender interactions for cardiovascular health. We employed welch two-sample t-tests and chi-Squared tests to examine gender-specific variations among predictor variables, enabling the identification of gender differences in risk factors which may not be evident in aggregated analyses. These results are included in the descriptives table.

ECog was treated as an outcome variable in a series of linear regression models. The first model (Model 1) examined demographic and psychosocial associations with ECog score. This included age, gender, years of education, race, ethnicity, GDS Score, quality of health and quality of sleep as independent variables. A linear regression model including demographic only associations with ECog without psychosocial variables can be referenced in supplementary Table 2.

The second model (model 2) expanded upon the first model by incorporating self-reported cardiovascular conditions and risk factors including BMI, high blood pressure, high cholesterol, heart disease and diabetes and alcohol use or tobacco use. The final third model (model 3) included gender interactions for the cardiovascular health variables. The results are reported as effect sizes, unstandardised regression coefficients, 95% confidence intervals, and p-values.

Effect sizes for continuous, numeric or binary variables are in Cohen’s D. Effect sizes for multilevel categorical variables, race and BMI, are reported as Cohen’s f2 (η^2^). Cohen’s D and Cohen’s f2 (η^2^) have not been adjusted for any other covariates or predictors in the model. Both effect sizes used in this paper provide a measure of the strength of association between the dependent variable and ECog alone, without considering other factors in the model. All p-values correspond to regression coefficients. R^2^ values are reported for each model to observe whether additional categories of predictors were able to show explanatory power.

## Results

### Participants

Table 1 shows descriptive statistics of the included cohort, stratified by gender. Welch two sample t-tests were used to examine whether there were statistically significant differences between genders for continuous variables, and chi-squared was used for dichotomous variables. Males (n=6,600) had a higher mean age of 71.33 years compared with females (n=20,996) with a mean age of 68.71 years. Males reported more years of education (mean=16.78) than females (mean=16.12). There was no significant difference in the mean ECog score between males and females. The percentage of African American individuals (total n=692) was lower among males (n=109; 1.7%) compared with females (n=583; 2.8%), similarly, Native American identification was lower among males (n=135; 2.1%) compared with females (n=579; 2.8%). Females also had a higher representation of Latino individuals (n=2,995; 14%) compared with males (n=580; 8.8%). There were a higher percentage of Asian males (n=221; 3.4%) compared with females (n=557; 2.7%).

**Table 1:** Descriptive Statistics and Gender Differences in Study Variables

**Variable**	**Male**	**Female**	**Total**
Total N	6,600 (24%)	20,996 (76%)	27,596
Age (years)			
Mean, SD	71.33 (8.28)	68.71 (7.77)	69.36, 7.98 †
Min – Max	55 – 92	55 – 91	
Years of Education			
Mean, SD	16.78 (2.36)	16.12 (2.32)	16.27 (2.35) †
Min – Max	12 – 20	12 –20	
ECog score			
Mean, SD	1.44 (0.49)	1.44 (0.47)	1.44 (0.47)
Min – Max	1 – 4	1 – 4	
African American			
N, % total	109 (1.7%)	583 (2.8%)	692 (2.5%) *
Asian			
N, % total	221 (3.4%)	557 (2.7%)	778 (2.8%) *
White			
N, % total	5,802 (87.7%)	18,240 (87%)	24,078 (87%)
Latino			
N, % total	580 (8.8%)	2,995 (14%)	3,581 (13%) *
Native American			
N, % total	135 (2.1%)	579 (2.8%)	719 (2.6%) *
Pacific Islander			
N, % total	25 (0.4%)	77 (0.4%)	104 (0.4%)
More than one race			
N, % total	182 (2.8%)	539 (2.6%)	721 (2.6%)
Family history of AD			
N, % total	1,965 (30%)	7,053 (33.5%)	9,032 (33%)
GDS score			
Mean, SD	2.83 (3.16)	3.25 (3.31)	3.15 (3.30) †
Min - Max	0 – 12	0 – 14	
Quality of Health			
N, % total good health	4,729 (72%)	14,719 (70%)	19,448 (70.5%)
Quality of Sleep			
N, % total good sleep	2,897 (44%)	8,079 (39%)	10,976 (40%) *
Alcohol or tobacco use			
N, % total	552 (8.4%)	970 (4.6%)	1,522 (5.5%)
BMI			
underweight %, normal %, overweight %	0.4%, 19%, 80%	0.7%, 44%, 55%	0.6%, 39%, 60%
High blood pressure			
N, % total	2,169 (33%)	5,011 (24%)	7,180 (26%) *
High cholesterol			
N, % total	374 (5.7%)	904 (4.3%)	1,278 (4.6%) *
Diabetes			
N, % total	525 (8%)	1,309 (6.2%)	1,840 (6.6%) *
Heart disease			
N, % total	707 (11%)	879 (4.2%)	1,586 (5.6%) *

Note. † = Welch two sample t-test p <0.001 different to male, * = Chi Squared p<0.01 different to male.

Females had higher GDS scores, indicating more depressive symptoms. Self-reported good quality of sleep was higher in males (44% good sleep) compared with females (39% good sleep). No differences were found in alcohol and tobacco use between males and females. The distribution of BMI categories differed between males and females. Among males, 19% fell into the normal BMI range and 80% were classified as overweight. Among females, 44% had a normal BMI and 55% were classified as overweight. The prevalence of high blood pressure, high cholesterol, diabetes and heart disease was higher among males compared with females (Table 1). Univariable models for associations of each variable of interest with ECog are in (Supplemental Table 1).

### Associations of ECog Score with Demographic and Psychosocial Variables (Table 2)

Table 2 presents model 1: the results of the first multivariable linear regression model to assess associations with SCD and demographic and psychosocial variables. The following variables were associated with higher self-reported SCD (ECog): higher age (d = 0.002, p < 0.001), male gender (d = −0.001, p = 0.009), higher GDS score (d = 0.06, p < 0.001), poorer quality of health (d = -0.42, p<0.001) and poorer sleep quality (d = −0.18, p < 0.001). No associations were found between race or ethnicity and ECog scores. R^2^ = 0.19.

**Table 2:** Associations of ECog Score with Demographic and Psychosocial Predictors

	**Effect Size**	**ECog**		
		**Unstandardised Regression Coefficient**	**95% Confidence Interval**	**P-Value**
Age (years)	0.002	0.003	0.002, 0.004	<0.001
Gender (0=male)	−0.001	−0.03	−0.04, −0.01	0.009
Years of education	−0.02	−0.001	−0.003, 0.004	0.85
Race (ref = White)				
African American	0.08	−0.06	−0.13, 0.008	0.08
Asian		0.04	−0.01, 0.10	0.12
Native American		0.08	−0.09, 0.25	0.37
Pacific Islander		−0.09	−0.45, 0.25	0.60
More than one race		0.05	−0.06, 0.17	0.66
Ethnicity				
Latino	0.12	0.02	−0.04, 0.08	0.55
Family history of AD	0.49	0.01	−0.004, 0.03	0.13
GDS score	0.06	0.06	0.05, 0.06	<0.001
Health (0= bad, 1=good)	−0.42	−0.19	−0.22, −0.17	<0.001
Quality of Sleep (0= bad, 1=good)	−0.18	−0.06	−0.08, −0.04	<0.001

Note. Effect size for continuous and binary variables is Cohen’s D. Effect size for multilevel categorical variables, race and BMI is Cohen’s f2 (η^2^). R^2^=0.19.

### Contributions of Cardiovascular Risk Factors and Medical Conditions (Table 3)

Introducing cardiovascular risk factors and related medical conditions in model 2 (Table 3) in addition to demographic and psychosocial factors showed associations remained statistically significantly associated for higher ECog and advancing age (p<0.001), higher GDS (p<0.001), poorer self-reported health (p<0.001) and poorer self-reported sleep quality (p<0.001). Higher ECog score was additionally associated with use of alcohol and tobacco (d = 0.15, p=0.01). Obese BMI was negatively associated with ECog scores when compared with normal BMI (unstandardised regression coefficient = −0.03, p = 0.01). No associations were found between race or ethnicity and ECog scores. The R^2^ value presented in model 2 including cardiovascular conditions and risk factors (Table 3) showed no change from the previous model in (Table 2) (R^2^ = 0.19).

**Table 3:** Associations of ECog with Demographics, Psychosocial and Cardiovascular Risk Factors

	**Effect Size**	**ECog**		
		**Unstandardised Regression Coefficient**	**95% Confidence Interval**	**P-Value**
Age (years)	0.002	0.003	0.001, 0.004	<0.001
Gender (0=male)	−0.001	−0.01	−0.03, 0.006	0.20
Years of education	−0.02	0.001	−0.002, −0.005	0.51
Race				
African American	0.08	−0.06	−0.13, 0.01	0.11
Asian		0.05	−0.004, 0.11	0.06
Native American		0.08	−0.1, 0.25	0.38
Pacific Islander		−0.09	−0.44, 0.26	0.60
More than one race		0.04	−0.07, 0.16	0.68
Ethnicity				
Latino	0.12	0.02	−0.04, 0.08	0.53
Family history of AD	0.49	0.01	−0.004, 0.03	0.14
GDS score (0 – 14)	0.06	0.05	0.05, 0.06	<0.001
Quality of Health	−0.42	−0.19	−0.23, −0.17	<0.001
Quality of Sleep (0= bad, 1=good)	−0.18	−0.06	−0.08, −0.04	<0.001
Alcohol or tobacco use	0.15	0.04	0.007, 0.07	0.01
Underweight BMI (comparison group normal)	0.10	0.07	−0.02, 0.17	0.15
Overweight BMI (comparison group normal)		−0.02	−0.04, 0.003	0.09
Obese BMI (comparison group normal)		−0.03	−0.05, −0.006	0.01
High Blood pressure	0.08	0.004	−0.01, 0.03	0.65
Cholesterol	0.16	0.04	−0.03, 0.11	0.21
Diabetes	−0.15	−0.03	−0.07, 0.04	0.37
Heart disease	0.10	0.03	−0.02, 0.06	0.05

Note. Effect size for continuous and binary variables is Cohen’s D. Effect size for multilevel categorical variables, race and BMI is Cohen’s f2 (η^2^). R^2^=0.19.

### Effects of Gender Interactions with Cardiovascular Risk Factors and Conditions

Model 3 (Table 4) introduces gender interactions with cardiovascular risk factors and conditions. In this model higher ECog score was associated with advancing age, GDS Score, poorer self-reported quality of health, poorer sleep quality and underweight BMI (unstandardised regression coefficient = 0.32, p = 0.02).

**Table 4:** Gender Interactions with Cardiovascular Predictors of ECog Score

	**Effect Size**	**ECog and gender interactions**		
		**Unstandardised Regression Coefficient**	**95% Confidence Interval**	**P-Value**
Age (years)	0.002	0.002	0.001, 0.004	<0.001
Gender (0=male)	−0.001	−0.03	−0.07, 0.003	0.07
Years of Education	−0.02	0.001	−0.002, 0.05	0.51
Race				
African American	0.08	−0.06	−0.13, 0.01	0.10
Asian		0.05	−0.005, 0.11	0.07
Native American		0.08	−0.09, 0.25	0.39
Pacific Islander		−0.09	−0.45, 0.26	0.59
More than one race		0.04	−0.09, 0.18	0.67
Ethnicity				
Latino	0.12	0.02	−0.04, 0.07	0.56
Family history of AD	0.49	0.0	−0.004, 0.03	0.13
GDS Score (0 – 14)	0.06	0.05	0.05, 0.06	<0.001
Quality of Health	−0.42	−0.20	−0.23, −0.17	<0.001
Sleep quality (0= bad, 1=good)	−0.18	−0.06	−0.08, −0.04	<0.001
Alcohol or tobacco use	0.15	0.01	−0.06, 0.04	0.76
Underweight BMI (comparison group normal)	0.10	0.32	0.06, 0.59	0.02
Overweight BMI (comparison group normal)		−0.03	−0.07, 0.01	0.11
Obese BMI (comparison group normal)		−0.02	−0.06, 0.02	0.35
High Blood Pressure	0.08	−0.01	−0.06, 0.18	0.50
Cholesterol	0.16	0.06	−0.06, 0.17	0.36
Diabetes	−0.15	−0.04	−0.14, 0.07	0.50
Heart Disease	0.10	0.22	−0.02, 0.07	0.33
Alcohol or tobacco use * Gender	0.07	0.08	0.01, 0.15	0.02
Underweight BMI * Gender		−0.29	−0.57, −0.01	0.04
Overweight BMI * Gender		0.02	−0.02, 0.06	0.34
Obese BMI * Gender		−0.01	−0.06, 0.04	0.67
High Blood Pressure * Gender		0.02	−0.02, 0.06	0.27
Cholesterol * Gender	−0.03	−0.02	−0.16, 0.13	0.82
Diabetes * Gender	0.02	0.01	−0.11, 0.14	0.84
Heart Disease * Gender	0.05	0.02	−0.04, 0.08	0.51

Note. Effect size for continuous and binary variables is Cohen’s D. Effect size for multilevel categorical variables, race and BMI is Cohen’s f2 (η^2^).

While the associations between higher Ecog and high blood pressure, high cholesterol and heart disease were more pronounced in females, there were no significant interactions between these medical conditions and gender (Table 4). However, a statistically significant interaction by gender was observed between use of alcohol or tobacco (d = −0.07, p=0.28) and underweight BMI and gender (unstandardised regression coefficient = −0.29, p = 0.04). Specifically, males who reported alcohol and tobacco use or underweight BMI had higher Ecog scores compared with females.

## Discussion

### Main Findings

Contrary to our hypotheses, when comparing race groups with the reference group (self-identify only as white), our analysis revealed no significant associations between SCD and self-identified race or with ethnicity. We found, as hypothesised, that presence of risk factors for poorer psychosocial health such as bad sleep quality, depressive symptoms and bad self-reported quality of health were associated with greater SCD (higher ECog scores). However, risk factors for poorer cardiovascular health such as high BMI and presence of medical conditions such as high blood pressure, high cholesterol, diabetes and heart disease were not associated with higher ECog scores. Alcohol and tobacco use was consistently associated with greater SCD. Finally, we found when examining gender interactions for cardiovascular conditions and risk factors that higher SCD levels were associated with alcohol and tobacco use in males but not in females and underweight BMI was also associated with higher SCD in males but not in females. In line with existing literature, our study demonstrated that older adults experiencing SCD were more likely to have an increased number of risk factors including depressive symptoms, lower quality of health, lower quality of sleep and alcohol or tobacco use. Importantly, our findings replicate previous findings within a remote cohort, supporting the robustness of the observed associations.

Older individuals and males exhibited higher ECog scores, indicating more self-reported decline. Age is a well-documented risk factor for declining cognitive health and this finding is in line with existing literature, however, the finding of higher ECog scores in male gender is noteworthy considering previous studies indicate a higher prevalence of SCD in women (16, 44). Higher prevalence of SCD in women has been attributed to biological factors such as menopause-related cognitive impairment influenced by estrogen-regulated systems like sleep, circadian rhythms, or within specific cognitive domains (45). This finding in our study may differ from past findings due to an unbalanced representation of men and women in the BHR cohort which raises the possibility that men with concerns may be more likely to join the BHR.

### Race and Ethnicity Associations with SCD

We found no associations of self-reported race or ethnicity identity with ECog scores in this study contrary to our hypotheses that differences in SCD between different racial or ethnic groups will be observed. Previous studies have identified depression and anxiety as predictors of SCD differences in Black and Hispanic groups (28). Our study did not identify variations in level of SCD among ethnoracial groups, nor did we reveal stronger associations of risk factors with SCD for African American, Asian, Native American or Pacific Islander self-report groups compared to self-reported white. It is essential to emphasise that the absence of discernible differences may be a consequence of methodological limitations, particularly in our use of a white reference group. The practice of using data from white individuals as a benchmark or control group, which assumes cultural equivalence, has faced criticism in recent years as selecting a culturally dominant group as the reference can subtly reinforce the notion that dominant groups are the most “normal”. Recommendations include using theory to inform hypotheses of differences, including mediators to explain group differences, and using mixed methods to reveal heterogeneity within groups.

### Psychosocial Associations with SCD

Depressive symptoms were consistently associated with higher ECog scores within all models, corroborating existing literature (46, 47). Subjective reports of lower sleep quality and quality of health were also found to be associated with higher ECog scores. These findings highlight the importance of sleep, quality of health and depressive symptoms as modifiable risk factors in the experience of SCD, independently of some medical conditions and demographic factors. Although these findings agree with previous research, it is known that sleep disturbance is a symptom of depression (14) and subjective ratings of cognitive symptoms are also a symptom of depression (17). There is a potentially complex interplay among these emotional and social dimensions in the experience and reporting of cognitive decline. To gain a deeper understanding of the true risk factors, it may be valuable to explore temporal associations between these psychosocial and cognitive symptoms.

### Cardiovascular Health Associations with Ecog

The associations between age, gender, depressive symptoms, sleep, and quality of health with ECog remained consistent even after accounting for medical conditions, indicating associations that may not mediated by the self-reported cardiovascular risk factors and conditions included in this study.

Alcohol and tobacco use were consistently associated with higher self-reported decline, particularly for males. Studies examining gender differences in alcohol or tobacco consumption have reported that rates of alcohol use disorder have been greater in men when compared to women, however, this gap is closing (48). It is possible that the current ageing population may present with consequences of alcohol use that reflect historic differences in the prevalence and amount of alcohol consumption between males and females i.e., men may present with higher rates of alcohol related problems compared with women.

Similarly, underweight BMI was associated with higher ECog scores in males when examining gender interactions. BMI has been strongly associated with cardiovascular risk factors such as decreased glucose tolerance, reduced insulin sensitivity and adverse lipid profiles (49–52). However, it is worth noting that BMI is considered an imperfect measure of health, criticised for its inaccuracy when used without considering waist-hip ratio and as a proxy for adiposity (49, 51, 53). This is particularly relevant for older adults, as changes in body composition with ageing can result in a lower BMI despite a higher proportion of body fat (54, 55). One study examining adiposity and cognitive decline in an elderly population found that higher adiposity was associated with better cognitive performance with similar results when excluding those with cardiovascular conditions, and when using waist circumference, BMI and fat-free mass as predictors (56). Higher adiposity may be protective for cognition in older adults, supported by evidence that weight loss can precede dementia diagnosis (56). This observation raises questions about the complex interplay between body composition, cognitive health, and the aging process.

Lastly, no significant associations were found for high blood pressure, diabetes, or high cholesterol. However, it is important to note that the cohort examined in this study is healthier than the general population on average in a number of ways. Notably, the prevalence of diabetes affects 11% of the US population (57), while only 6.6% of our study sample reported this condition. Similarly, hypertension is found in 48% of the US population (58), compared to 26% in our sample, and high cholesterol affects 10% of the US population (59), but only 4.6% of our study participants. Heart disease is slightly more prevalent in our study (5.6%) than in the general population60 (5%). Null findings regarding cardiovascular health markers could additionally be attributed to selection bias stemming from participants’ self-selection tendencies towards a healthier-than-average and health-conscious population, often referred to as the worried well.

### Limitations

Study limitations include a lack of balance between male and female groups, relatively small sample sizes for some sub-groups (e.g., n=104 for Pacific Islander identification, n=692 for African American identification), reliance on self-reported data instead of clinically-confirmed measures, and a substantial amount of unexplained variance, suggesting other factors beyond the included variables that contribute to SCD. 81% unaccounted variance suggests other factors, such as genetic, environmental, or psychosocial factors, or their complex interactions were not captured in the cross-sectional modelling approach. Further research incorporating a more comprehensive inclusion of predictors is needed to understand the complex interplay of factors contributing to SCD. It is worth emphasising when scrutinising remotely collected, self-reported data concerning demographic and psychosocial factors associated with cognitive health, encountering small coefficients and limited levels of explained variance is expected, and 19% of variance explained in context may still be informative, particularly as large sample size allows for power to distinguish small effects from noise. Additionally, as ECog can be a nonlinear metric, small movements in ECog response may still be indicative of meaningful differences in SCD self-assessment.

Although using education as a metric to indicate socioeconomic status may offer insights into various cognitive and lifestyle factors, it may not fully capture the nuanced and multifaceted nature of socioeconomic disparities. For example, individuals with similar educational backgrounds can experience significant variations in income, wealth, and living conditions. Additionally, using education as a sole indicator may overlook other key elements that contribute to SES, such as occupation, employment status, and family wealth. This oversimplification can mask important distinctions, leading to an incomplete understanding of the socioeconomic factors influencing health outcomes. Additionally, temporal changes cannot be considered when using education as a proxy for SES as economic and social dynamics evolve over time, and relying on education as a fixed metric may not accurately reflect an individual’s socioeconomic conditions as an older adult.

The lack of representation of historically under-included ethnocultural groups is a significant limitation that restricts generalisability and increases the risk of type 2 errors. Additionally, current approaches to reporting race and ethnicity tend to overlook nuances and differences based on geographic origins, cultural norms, language, as well as other historical factors with the consideration in study design that race and ethnicity are socially constructed classifications 61. To fill gaps in our understanding of the role of race and ethnicity in cognitive health, a more nuanced approach is needed, capturing the complexities of race and ethnicity as social determinants of health. With the growing success of remote studies, there is an opportunity for remote cohorts to be representative of the population, and include more underrepresented individuals. The BHR has explored recruitment strategies for online studies as an avenue towards a more diverse participant pool (38, 39). The registry has implemented features to increase enrolment of underrepresented populations, which have resulted in the enrolment of 7,013 individuals from underrepresented ethnocultural populations. This online approach may alter the composition of the study population compared with in person studies by limiting participation through selection bias as online research studies tend to demonstrate biases towards individuals with digital devices, internet access, and digital literacy. However, this approach increases research participation and accessibility for individuals encountering challenges in participating in in-person studies due to geographical constraints, time limitations and social anxiety.

Additionally, although participants were excluded if they self-reported AD, Dementia, Frontotemporal Dementia, Lewy Body Dementia, MCI, Multiple Sclerosis, or traumatic brain injury, we cannot rule out the possibility that our sample includes cognitively impaired participants. Lack of information on MCI status may have inadvertently led to the inclusion of cognitively impaired individuals as unimpaired. Depending solely on self-reported dementia may also be particularly problematic for individuals with impaired awareness, and reliance on this information may compromise the reliability of other self-reported data.

## Conclusion

This study identified demographic, psychosocial, and cardiovascular conditions associated with SCD in the BHR, a large, remote cohort of older adults. We demonstrated that age, quality of health, quality of sleep and alcohol or tobacco use were robustly associated with experience of cognitive decline. Additionally, this study demonstrated the value of remotely collected self-reported metrics in understanding demographic and health risk associations.

## Supplementary

Supplementary material, approximately 21.9 KB.

## Notes

*Ethical standards:* The authors of the study agree upon standards of expected ethical behavior for all parties involved.

## References

[1] Jessen F, Amariglio RE, Boxtel M, Breteler M, Ceccaldi M, Chételat G (2014). A conceptual framework for research on subjective cognitive decline in preclinical Alzheimer’s disease. Alzheimers Dement.

[2] Garcia-Ptacek S, Eriksdotter M, Jelic V, Porta-Etessam J, Kåreholt I, Palomo SM. Subjective cognitive impairment: Towards early identification of Alzheimer disease. 10.1016/j.nrl.2013.02.007. 10.1016/j.nrl.2013.02.007 23601758

[3] Lin Y, Shan PY, Jiang WJ, Sheng C, Ma L (2019). Subjective cognitive decline: preclinical manifestation of Alzheimer’s disease. Neurol Sci.

[4] Rueda AD, Lau KM, Saito N, Harvey D, Risacher SL, Aisen PS (2015). Self-rated and informant-rated everyday function in comparison to objective markers of Alzheimer’s disease. Alzheimers Dement.

[5] Tomaszewski Farias S, Mungas D, Harvey DJ, Simmons A, Reed BR, DeCarli C (2011). The measurement of everyday cognition: Development and validation of a short form of the Everyday Cognition scales. Alzheimers Dement.

[6] Tomaszewski Farias S, Mungas D, Reed BR, Cahn-Weiner D, Jagust W, Baynes K (2008). The measurement of everyday cognition (ECog): Scale development and psychometric properties. Neuropsychology.

[7] Ferraro KF, Sauerteig-Rolston MR, Barnes LL, Friedman E, Sands LP, Thomas PA (2023). Subjective Memory Decline Predicts Incident Cognitive Impairment Among White—But Not Black or Hispanic—Older Adults. Meeks S, editor. The Gerontologist.

[8] Gupta S (2021). Racial and ethnic disparities in subjective cognitive decline: a closer look, United States, 2015–2018. BMC Public Health.

[9] Hao L, Wang X, Zhang L, Xing Y, Guo Q, Hu X, et al. Prevalence, Risk Factors, and Complaints Screening Tool Exploration of Subjective Cognitive Decline in a Large Cohort of the Chinese Population. Zhu LQ, editor. J Alzheimers Dis. 2017 Sep 18;60(2):371–88. 10.3233/JAD-170347. 10.3233/JAD-170347 28869471

[10] Mazzeo S, Padiglioni S, Bagnoli S, Carraro M, Piaceri I, Bracco L (2020). Assessing the effectiveness of subjective cognitive decline plus criteria in predicting the progression to Alzheimer’s disease: an 11-year follow-up study. Eur J Neurol.

[11] Peterson RL, Carvajal SC, McGuire LC, Fain MJ, Bell ML (2019). State inequality, socioeconomic position and subjective cognitive decline in the United States. SSM - Popul Health.

[12] Abrams LR, Mehta NK (2019). Changes in depressive symptoms over age among older Americans: Differences by gender, race/ethnicity, education, and birth cohort. SSM - Popul Health.

[13] Berezuk C, Khan M, Callahan BL, Ramirez J, Black SE, Zakzanis KK (2023). Sex differences in risk factors that predict progression from mild cognitive impairment to Alzheimer’s dementia. J Int Neuropsychol Soc.

[14] Nutt D, Wilson S, Paterson L (2008). Sleep disorders as core symptoms of depression. Dialogues Clin Neurosci.

[15] Peter-Derex L, Yammine P, Bastuji H, Croisile B (2015). Sleep and Alzheimer’s disease. Sleep Med Rev.

[16] Roh M, Dan H, Kim O (2021). Influencing Factors of Subjective Cognitive Impairment in Middle-Aged and Older Adults. Int J Environ Res Public Health.

[17] Yates JA, Clare L, Woods RT, Matthews FE. Subjective Memory Complaints are Involved in the Relationship between Mood and Mild Cognitive Impairment. Tales A, Jessen F, Butler C, Wilcock G, Phillips J, Bayer T, editors. J Alzheimers Dis. 2015 Sep 24;48(s1):S115-23. 10.3233/JAD-150371. 10.3233/JAD-150371 26402102

[18] Omura JD, McGuire LC, Patel R, Baumgart M, Lamb R, Jeffers EM (2022). Modifiable Risk Factors for Alzheimer Disease and Related Dementias Among Adults Aged ≥45 Years — United States, 2019. MMWR Morb Mortal Wkly Rep.

[19] Dickie DA, Valdés Hernandez MDC, Makin SD, Staals J, Wiseman SJ, Bastin ME (2018). The brain health index: Towards a combined measure of neurovascular and neurodegenerative structural brain injury. Int J Stroke.

[20] España-Irla G, Gomes-Osman J, Cattaneo G, Albu S, Cabello-Toscano M, Solana-Sanchéz J (2021). Associations Between Cardiorespiratory Fitness, Cardiovascular Risk, and Cognition Are Mediated by Structural Brain Health in Midlife. J Am Heart Assoc.

[21] Kulshreshtha A, Goetz M, Alonso A, Shah AJ, Bremner JD, Goldberg J (2019). Association Between Cardiovascular Health and Cognitive Performance: A Twins Study. J Alzheimers Dis.

[22] Lamar M, Boots EA, Arfanakis K, Barnes LL, Schneider JA (2020). Common Brain Structural Alterations Associated with Cardiovascular Disease Risk Factors and Alzheimer’s Dementia: Future Directions and Implications. Neuropsychol Rev.

[23] Moroni F, Ammirati E, Rocca MA, Filippi M, Magnoni M, Camici PG (2018). Cardiovascular disease and brain health: Focus on white matter hyperintensities. IJC Heart Vasc.

[24] Wardlaw JM, Valdés Hernández MC, Muñoz-Maniega S (2015). What are White Matter Hyperintensities Made of?: Relevance to Vascular Cognitive Impairment. J Am Heart Assoc.

[25] Ebrahim S, Smith GD (1997). Systematic review of randomised controlled trials of multiple risk factor interventions for preventing coronary heart disease. BMJ.

[26] Majoka MA, Schimming C (2021). Effect of Social Determinants of Health on Cognition and Risk of Alzheimer Disease and Related Dementias. Clin Ther.

[27] Hill-Briggs F, Adler NE, Berkowitz SA, Chin MH, Gary-Webb TL, Navas-Acien A (2021). Social Determinants of Health and Diabetes: A Scientific Review. Diabetes Care.

[28] Robinson T, Klinger H, Buckley R, Deters KD, Quiroz YT, Rentz D (2023). Subjective cognitive decline across ethnoracial groups in the A4 study. Alzheimers Dement.

[29] Beam CR, Kaneshiro C, Jang JY, Reynolds CA, Pedersen NL, Gatz M (2018). Differences Between Women and Men in Incidence Rates of Dementia and Alzheimer’s Disease. J Alzheimers Dis.

[30] Brown MJ, Patterson R (2020). Subjective Cognitive Decline Among Sexual and Gender Minorities: Results from a U.S. Population-Based Sample. J Alzheimers Dis.

[31] Oliver MD, Morrison C, Kamal F, Graham J, Dadar M (2022). Subjective cognitive decline is a better marker for future cognitive decline in females than in males. Alzheimers Res Ther.

[32] Rexrode KM, Madsen TE, Yu AYX, Carcel C, Lichtman JH, Miller EC (2022). The Impact of Sex and Gender on Stroke. Circ Res.

[33] Gerdts E, Regitz-Zagrosek V (2019). Sex differences in cardiometabolic disorders. Nat Med.

[34] Peters SAE, Muntner P, Woodward M (2019). Sex Differences in the Prevalence of, and Trends in, Cardiovascular Risk Factors, Treatment, and Control in the United States, 2001 to 2016. Circulation.

[35] Ferretti MT, Iulita MF, Cavedo E, Chiesa PA, Schumacher Dimech A, Santuccione Chadha A (2018). Sex differences in Alzheimer disease — the gateway to precision medicine. Nat Rev Neurol.

[36] Mehta LS, Velarde GP, Lewey J, Sharma G, Bond RM, Navas-Acien A (2023). Cardiovascular Disease Risk Factors in Women: The Impact of Race and Ethnicity: A Scientific Statement From the American Heart Association. Circulation.

[37] Weiner MW. The BrainHealthRegistry.org: Using the Internet for identification, assessment, screening, recruitment, and longitudinal monitoring of subjects for neuroscience and Alzheimer’s disease studies. J Prev Alzheimers Dis. 2014;1-3. 10.14283/jpad.2014.9. 10.14283/jpad.2014.9 PMC5469285 28620591

[38] Weiner MW, Nosheny R, Camacho M, Truran-Sacrey D, Mackin RS, Flenniken D (2018). The Brain Health Registry: An internet-based platform for recruitment, assessment, and longitudinal monitoring of participants for neuroscience studies. Alzheimers Dement.

[39] Weiner MW, Aaronson A, Eichenbaum J, Kwang W, Ashford MT, Gummadi S, et al. Brain health registry updates: An online longitudinal neuroscience platform. Alzheimers Dement. 2023 Apr 18;alz.13077. 10.1002/alz.13077. 10.1002/alz.13077 PMC10518371 36965096

[40] Ashford MT, Neuhaus J, Jin C, Camacho MR, Fockler J, Truran D, et al. Predicting amyloid status using self-report information from an online research and recruitment registry: The Brain Health Registry. Alzheimers Dement Diagn Assess Dis Monit [Internet]. 2020 Jan [cited 2023 Aug 29];12(1). Available from: https://onlinelibrary.wiley.com/doi/10.1002/dad2.12102, 10.1002/dad2.12102. 10.1002/dad2.12102 PMC7513627 33005723

[41] Mackin RS, Insel PS, Truran D, Finley S, Flenniken D, Nosheny R (2018). Unsupervised online neuropsychological test performance for individuals with mild cognitive impairment and dementia: Results from the Brain Health Registry. Alzheimers Dement Diagn Assess Dis Monit.

[42] Almeida OP, Almeida SA (1999). Short versions of the geriatric depression scale: a study of their validity for the diagnosis of a major depressive episode according to ICD-10 and DSM-IV. Int J Geriatr Psychiatry.

[43] Yesavage JA, Brink TL, Rose TL, Lum O, Huang V, Adey M (1982). Development and validation of a geriatric depression screening scale: A preliminary report. J Psychiatr Res.

[44] Taylor CA, Bouldin ED, McGuire LC (2018). Subjective Cognitive Decline Among Adults Aged ≥45 Years — United States, 2015–2016. MMWR Morb Mortal Wkly Rep.

[45] Morgan KN, Derby CA, Gleason CE (2018). Cognitive Changes with Reproductive Aging, Perimenopause, and Menopause. Obstet Gynecol Clin North Am.

[46] Byers AL, Yaffe K (2011). Depression and risk of developing dementia. Nat Rev Neurol.

[47] Chu CS, Sun IW, Begum A, Liu SI, Chang CJ, Chiu WC (2017). The association between subjective memory complaint and objective cognitive function in older people with previous major depression. Homberg J, editor. PLOS ONE.

[48] Peltier MR, Verplaetse TL, Mineur YS, Petrakis IL, Cosgrove KP, Picciotto MR (2019). Sex differences in stress-related alcohol use. Neurobiol Stress.

[49] Buss J. Limitations of Body Mass Index to Assess Body Fat. 2014; 10.1177/216507991406200608. 10.1177/216507991406200608 24971823

[50] Dalton M, Cameron AJ, Zimmet PZ, Shaw JE, Jolley D, Dunstan DW (2003). Waist circumference, waist-hip ratio and body mass index and their correlation with cardiovascular disease risk factors in Australian adults. J Intern Med.

[51] Huxley R, Mendis S, Zheleznyakov E, Reddy S, Chan J (2010). Body mass index, waist circumference and waist:hip ratio as predictors of cardiovascular risk—a review of the literature. Eur J Clin Nutr.

[52] Ke JF, Wang JW, Lu JX, Zhang ZH, Liu Y, Li LX (2022). Waist-to-height ratio has a stronger association with cardiovascular risks than waist circumference, waist-hip ratio and body mass index in type 2 diabetes. Diabetes Res Clin Pract.

[53] Rothman KJ (2008). BMI-related errors in the measurement of obesity. Int J Obes.

[54] Woo J, Leung J, Kwok T (2007). BMI, Body Composition, and Physical Functioning in Older Adults*. Obesity.

[55] DeCaria JE, Sharp C, Petrella RJ (2012). Scoping review report: obesity in older adults. Int J Obes.

[56] Luchsinger JA, Biggs ML, Kizer JR, Barzilay J, Fitzpatrick A, Newman A (2013). Adiposity and Cognitive Decline in the Cardiovascular Health Study. Neuroepidemiology.

[57] Centers for Disease Control and Prevention. National Diabetes Statistics Report website. https://www.cdc.gov/diabetes/data/statistics-report/index.html. Accessed 20 December 2023.

[58] Wall HK, Ritchey MD, Gillespie C, Omura JD, Jamal A, George MG (2018). Vital Signs: Prevalence of Key Cardiovascular Disease Risk Factors for Million Hearts 2022 — United States, 2011–2016. MMWR Morb Mortal Wkly Rep.

[59] Heart Disease and Stroke Statistics—2023 Update: A Report From the American Heart Association. Circ 2023147e93-e621. 10.1161/CIR.0000000000001123 PMC12135016 36695182

[60] Tsao CW, Aday AW, Almarzooq ZI, Alonso A, Beaton AZ, Bittencourt MS, et al. Heart Disease and Stroke Statistics—2022 Update: A Report From the American Heart Association. Circulation [Internet]. 2022 Feb 22 [cited 2023 Dec 6];145(8). Available from: https://www.ahajournals.org/doi/10.1161/CIR.0000000000001052, 10.1161/CIR.0000000000001052. 10.1161/CIR.0000000000001052 35078371

[61] Flanagin A. Updated Guidance on the Reporting of Race and Ethnicity in Medical and Science Journals. AMWA J [Internet]. 2021 [cited 2023 May 19];38(1). Available from: https://amwajournal.org/index.php/amwa/article/view/195, 10.55752/amwa.2023.195. 10.1001/jama.2021.13304 34402850

