# Supplementary material for: Examining Demographic Factors, Psychosocial Wellbeing and Cardiovascular Health in Subjective Cognitive Decline in the Brain Health Registry Cohort
Source: J Prev Alzheimers Dis. 2024 Mar 5;11(3):787–97. doi: 10.14283/jpad.2024.39 (PMC11061024; doi:10.14283/jpad.2024.39)
Supplement: Supplementary file 1 — Supplementary material, approximately 21.9 KB. [file 42414_2024_327_MOESM1_ESM.docx]

## **Supplementary**

**Supplementary Table 1. Individual Predictor Associations with ECog Score.**

|  |  | **ECog** | | |
| --- | --- | --- | --- | --- |
|  | **Effect Size** | **Unstandardised Regression Coefficient** | **95% Confidence Interval** | **P-Value** |
| **Age (years)** | **0.002** | **-0.002** | **-0.003, 0.001** | **<0.001** |
| Gender (0=male) | -0.001 | -0.001 | -0.01, -0.012 | 0.88 |
| **Years of education** | **-0.02** | **-0.022** | **-0.024, -0.02** | **<0.001** |
| Race |  |  |  |  |
| African American | **0.08** | -0.02 | -0.05, 0.02 | 0.34 |
| Asian |  | -0.02 | -0.06, 0.16 | 0.25 |
| **Native American** |  | **0.16** | **0.10, 0.22** | **<0.001** |
| Pacific Islander |  | 0.11 | -0.03, 0.25 | 0.15 |
| More than one race |  | 0.07 | -0.09, 0.16 | 0.36 |
| Ethnicity |  |  |  |  |
| **Latino** | **0.12** | **0.12** | **0.10, 0.13** | **<0.001** |
| **Family history of AD** | **0.49** | **0.05** | **-0.04, 0.06** | **<0.001** |
| **GDS score (0 - 14)** | **0.06** | **0.06** | **0.05, 0.06** | **<0.001** |
| **Quality of Health** | **-0.42** | **-0.41** | **-0.43, -0.39** | **<0.001** |
| **Quality of Sleep (0= bad, 1=good)** | **-0.18** | **-0.18** | **-0.19, -0.16** | **<0.001** |
| **Alcohol or tobacco use** | **0.15** | **0.15** | **0.13, 0.17** | **<0.001** |
| **BMI (0=normal, 1=underweight)** | **0.10** | 0.02 | -0.01, 0.10 | 0.06 |
| **BMI (0=normal, 1=overweight)** |  | **0.02** | **0.001, 0.037** | **0.03** |
| **BMI (0=normal, 1=obese)** |  | **0.08** | **0.06, 0.06** | **<0.001** |
| **High Blood pressure** | **0.08** | **0.08** | **0.06, 0.03** | **<0.001** |
| **Cholesterol** | **0.16** | **0.16** | **0.13, 0.18** | **<0.001** |
| **Diabetes** | **-0.15** | **0.16** | **0.12, 0.17** | **<0.001** |
| **Heart disease** | **0.10** | **0.10** | **0.08, 0.13** | **<0.001** |

*Note. Table shows individual associations of each predictor with ECog in a single table. Effect size for continuous and binary variables is Cohen’s D. Effect size for multilevel categorical variables, race and BMI is Cohen's*f*2 (η²).*

**Supplementary Table 2. Demographic Variable Associations with ECog.**

|  |  | **ECog** | | |
| --- | --- | --- | --- | --- |
|  | **Effect size** | **Unstandardised regression coefficient** | **95% confidence interval** | **p-value** |
|  |  |  |  |  |
| **Age (years)** | **0.002** | **-0.0004** | **-0.001, 0.003** | **0.02** |
| **Gender (0=male)** | **-0.001** | **-0.02** | **-0.04, -0.01** | **<0.001** |
| Years of education | -0.02 | -0.02 | -0.02, -0.01 | **<0.001** |
| Race (reference group = White) |  |  |  |  |
| African American | **0.08** | -0.004 | -0.05, 0.04 | 0.85 |
| Asian |  | 0.001 | -0.04, 0.05 | 0.94 |
| **Native American** |  | **0.09** | **0.03, 0.16** | **<0.001** |
| Pacific Islander |  | -0.006 | -0.18, 0.17 | 0.95 |
| More than one race |  | 0.002 | -0.001, 0.06 | 0.78 |
| Ethnicity |  |  |  |  |
| Latino | **0.12** | **0.06** | **0.04, 0.09** | **<0.001** |

*Note. Effect size for continuous and binary variables is Cohen’s D. Effect size for multilevel categorical variables, race and BMI is Cohen's*f*2 (η²).*

**Supplementary Table 3. Comparison of BHR Cohort Descriptives by Individuals Included and Excluded.**

| **Variable** | **Excluded** | **Included** |
| --- | --- | --- |
| **Total N** | **62,633** | **27,596** |
| Age (years)  *Mean, SD* | 66.5 (7.47)  *(n=61,254)* | 69.36, 7.98  *(n=27,596)* |
| Gender  *N, % total female* | 45,716 (73%)  *(n=60,657)* | 20,996 (76%)  *(n=27,596)* |
| Years of Education  *Mean, SD* | 16 (2.38)  *(n=59,267)* | 16.27 (2.35)  *(n=27,596)* |
| ECog score  *Mean, SD* | 1.60 (0.81)  *(n=57,329)* | 1.44 (0.47)  *(n=27,596)* |
| African American  *N, % total* | 3,308 (5.3%) | 692 (2.5%) |
| Asian  *N, % total* | 2,360 (3.8%) | 778 (2.8%) |
| White  *N, % total* | 47,638 (76%) | 24,078 (87%) |
| Latino  *N, % total* | 8,421 (13%) | 3,581 (13%) |
| Native American  *N, % total* | 1,978 (3%) | 719 (2.6%) |
| Pacific Islander  *N, % total* | 325 (0.52%) | 104 (0.4%) |
| More than one race  *N, % total* | 2,663 (4.3%) | 721 (2.6%) |
| Family history of AD  *N, % total* | 9,950 (16%)  *(n=57,515)* | 9,032 (33%)  *(n=27,245)* |
| GDS score  *Mean, SD* | 2.61 (4.34)  *(n=57,290)* | 3.15 (3.30)  *(n=27,231)* |
| Quality of Health  *N, % total good health* | 29,418 (47%)  *(n=23,711)* | 19,448 (70.5%)  *(n=26,322)* |
| Quality of Sleep  *N, % total good sleep* | 21,608 (35%)  *(n=24,608)* | 10,976 (40%)  *(n=21,594)* |
| Alcohol or tobacco use  *N, % total* | 5,421 (8.8%)  *(n=61, 714)* | 1,522 (5.5%)  *(n=25,723)* |
| BMI  *underweight %, normal %, overweight %* | 1%, 37%, 62%  *(n=62,420)* | 0.6%, 39%, 60%  *(n=26,791)* |
| High blood pressure  *N, % total* | 11,535 (18%)  *(n=28,484)* | 7,180 (26%)  *(n=26,191)* |
| High cholesterol  *N, % total* | 2, 747 (4.4%)  *(n=28,484)* | 1,278 (4.6%)  *(n=26,191)* |
| Diabetes  *N, % total* | 3,188 (11.2%)  *(n=28,484)* | 1,840 (6.6%)  *(n=26,191)* |
| Heart disease  *N, % total* | 1,599 (5.6%)  *(n=28,484)* | 1,586 (5.6%)  *(n=26,191)* |
